# Laparoscopic and endoscopic cooperative surgery for gastrointestinal stromal tumor with complete situs inversus: report of a case

**DOI:** 10.1186/s40792-015-0076-7

**Published:** 2015-09-02

**Authors:** Mikito Mori, Kiyohiko Shuto, Atsushi Hirano, Chihiro Kosugi, Kuniya Tanaka, Keiji Koda

**Affiliations:** Department of Surgery, Teikyo University Chiba Medical Center, 3426-3 Anesaki, Ichihara, 299-0111 Japan

**Keywords:** Complete situs inverse, Gastrointestinal stromal tumor, Laparoscopic and endoscopic cooperative surgery

## Abstract

We herein report our experience of performing laparoscopic and endoscopic cooperative surgery for a gastrointestinal stromal tumor with complete situs inversus. A 78-year-old man was referred to our department for treatment of a gastric submucosal tumor. Based on chest X-ray and computed tomography (CT) findings, complete situs inversus was also diagnosed. Upper gastrointestinal endoscopy and imaging showed a 45-mm gastric submucosal tumor in the upper stomach near the esophagogastric junction. We performed local resection of the gastric submucosal tumor by laparoscopic and endoscopic cooperative surgery. Pathological examination revealed that the tumor was an intermediate-risk gastrointestinal stromal tumor, and the patient was discharged on postoperative day 12. The patient is still alive without recurrence or any complications 9 months after surgery.

## Background

Complete situs inversus (CSI), a relatively rare congenital condition, is found in one per 6000–8000 persons [1]. Some reports of patients with CSI undergoing surgery for gastric cancer have been published [2–6]. However, there have been few reports of non-epithelial malignancies associated with CSI [7]. Like CSI, gastrointestinal stromal tumors (GISTs), which are non-epithelial malignancies that mostly arise in the stomach, are rare. Because GISTs rarely involve lymph nodes, it is important to ensure histologically complete removal [8, 9]. Local resection is the standard treatment for GIST, and various laparoscopic techniques for achieving this have been developed [10–12]. Laparoscopic and endoscopic cooperative surgery (LECS), which was developed to facilitate precise dissection of gastrointestinal malignancies, is a form of minimally invasive surgery that can be used to resect GISTs [13]. To our knowledge, no cases of LECS for GIST associated with CSI have been reported. We herein report our experience of performing LECS for GIST with CSI.

## Case presentation

**Fig. 1:**
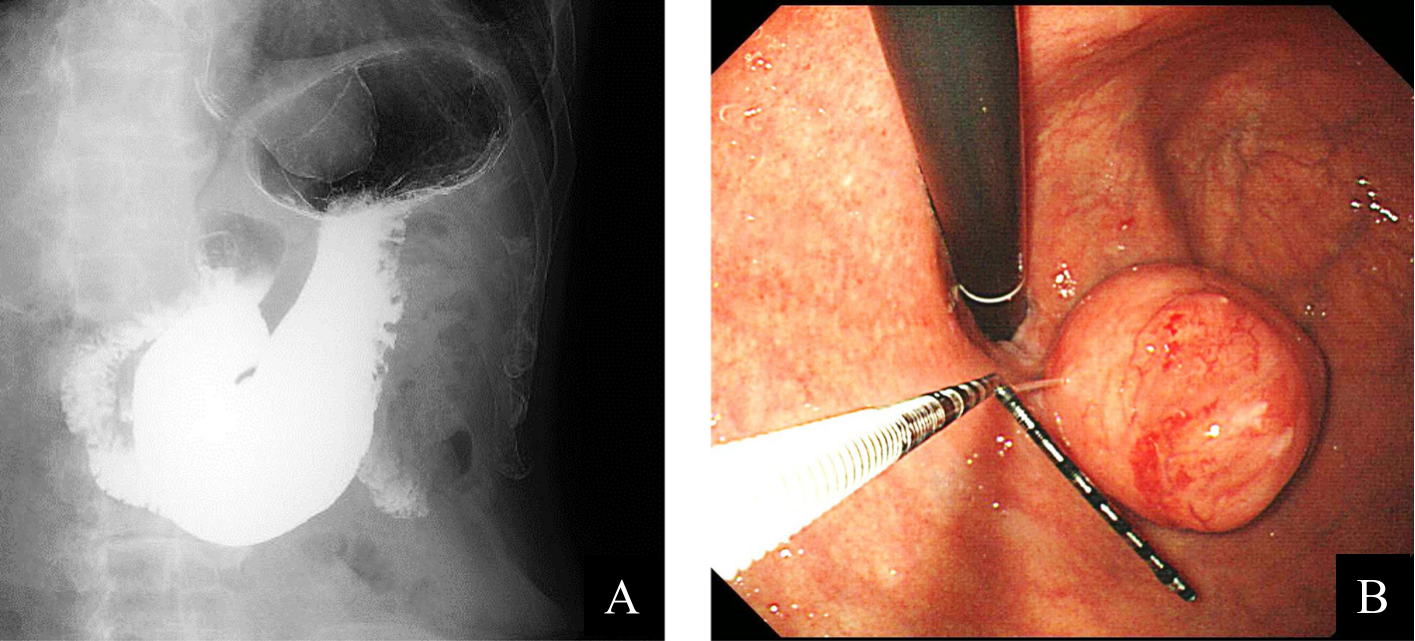
Upper gastrointestinal imaging (**a**) and endoscopy (**b**) of SMT. An SMT without mucosal defects in the upper stomach near the esophagogastric junction is demonstrated

**Fig. 2:**
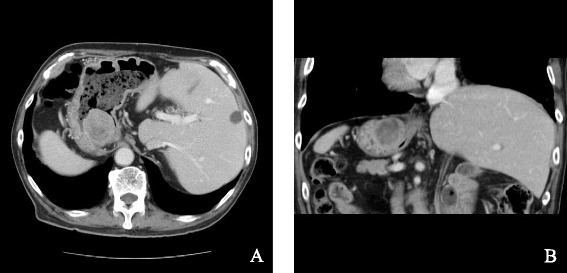
Abdominal CT images. A 45-mm tumor located in the upper area stomach is demonstrated; there is no evidence of distant metastasis. A hepatic cyst is also visible (**a**). All intra-abdominal organs are inversely positioned (**b**)

A 78-year-old man was referred to our department for treatment of a gastric submucosal tumor (SMT). Upper gastrointestinal endoscopy and imaging identified an SMT with no mucosal defects in the upper stomach near the esophagogastric junction (Fig. 1). A chest X-ray film showed dextrocardia. Abdominal computed tomography (CT) showed a 45-mm tumor in the upper stomach with no evidence of distant metastases, a hepatic cyst, and inverse positioning of all intra-abdominal organs (Fig. 2). Thus, the patient was diagnosed as having a gastric SMT associated with CSI, and local resection of the tumor was performed by LECS.

**Fig. 3:**
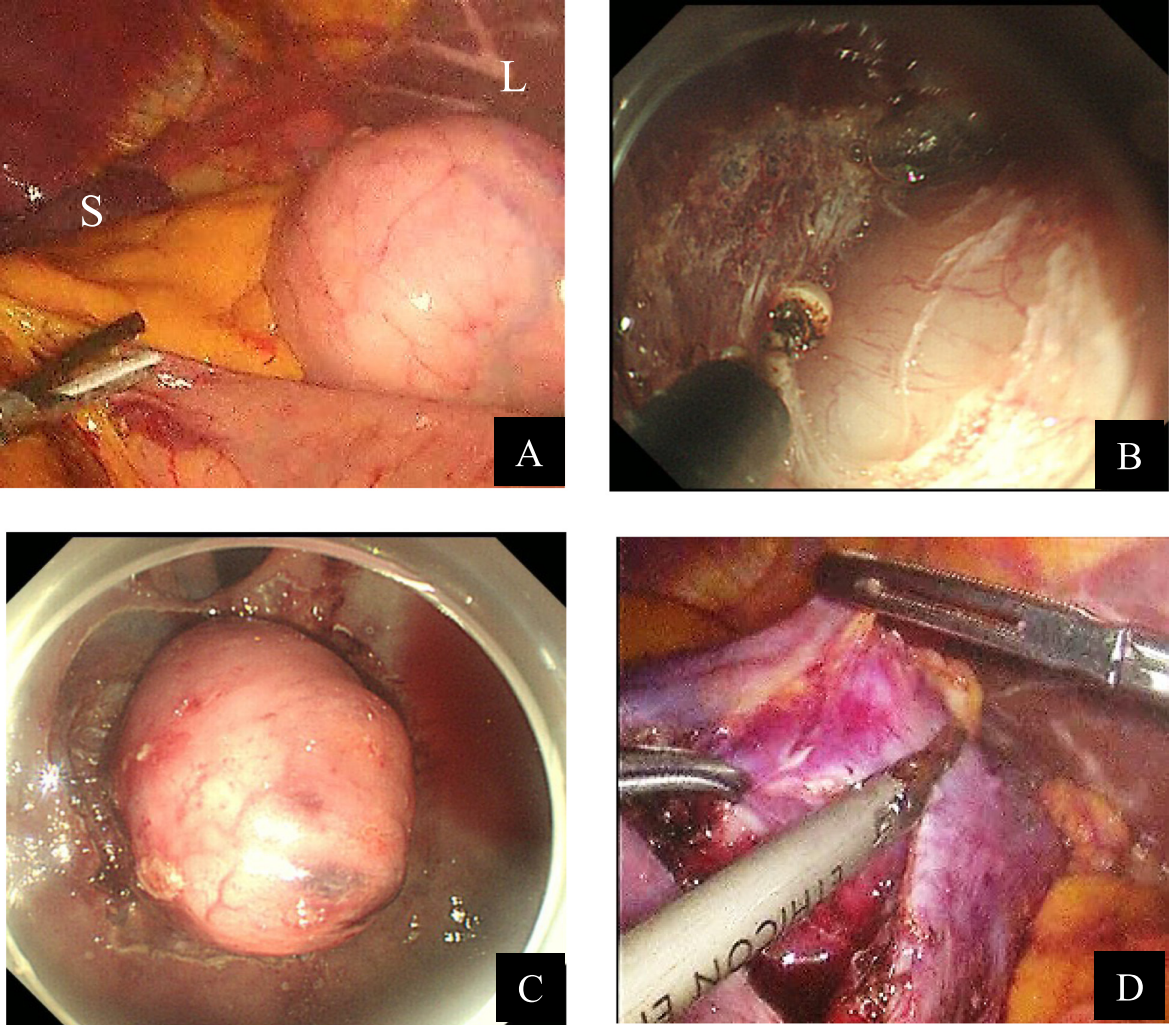
LECS procedure for dissection of the SMT. After the tumor location had been confirmed by intraluminal scope, blood vessels in the excision area around the tumor were minimally ligated using an ultrasonically activated device. The *letter S* indicates spleen, and the *letter of L* indicates liver (**a**). Endoscopic submucosal dissection was performed around the tumor (**b**). The seromuscular layer was intentionally perforated after three-quarters of the circumference of the excision had been finished (**c**). The tip of an ultrasonically activated device was inserted into the perforation and seromuscular dissection around the tumor performed (**d**)

A camera port was inserted through an umbilical incision using an open technique, after which four additional ports (two 5-mm ports and two 12-mm ports) were inserted into the left upper, left lower, right upper, and right lower quadrants, respectively, under a pneumoperitoneum of 10 mmHg. First, the tumor location was confirmed by intraluminal scope, after which the blood vessels in the excision area around the tumor were minimally ligated using an ultrasonically activated device. Next, endoscopic submucosal resection using endoscopic submucosal dissection (ESD) technique was performed around the tumor and the seromuscular layer was intentionally perforated after three-quarters of the circumference of the excision had been finished. The tip of an ultrasonically activated device was then inserted into the perforation and seromuscular dissection around the tumor performed. After the tumor had been resected and removed using a single-use specimen pouch, the incision line was closed using laparoscopic stapling devices (Fig. 3). The operating time was 220 min and blood loss of 455 mL.

**Fig. 4:**
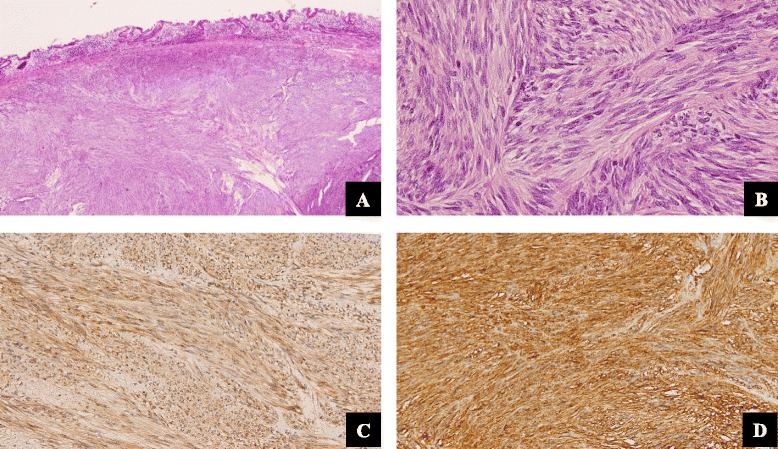
Histopathological findings. Histopathological examination showed that the tumor was composed of interlacing fascicles of spindle-shaped cells with elongated nuclei (hematoxylin and eosin stain, magnification ×20) (**a**), and the mitotic index was more than five mitotic figures per fifty high power fields (hematoxylin and eosin stain, magnification ×200) (**b**). Immunohistochemical analysis revealed that the tumor cells were positive for KIT (magnification ×100) (**c**) and CD34 (magnification ×100) (**d**)

Histopathological examination showed that the tumor was composed of interlacing fascicles of spindle-shaped cells with elongated nuclei; the mitotic index was more than five mitotic figures per fifty high power fields. Immunohistochemical analysis revealed that the tumor cells were positive for KIT and CD34 and negative for alpha-smooth muscle actin and S-100 (Fig. 4). The final diagnosis was intermediate-risk GIST according to the modified Fletcher classification [14]. Although blood transfusion was needed for postoperative anemia, the patient was discharged on postoperative day 12. He is still alive with no recurrence or complications 9 months after surgery.

### Discussion

CSI is a relatively rare congenital condition that occurs in one per 6000–8000 persons [1] and is associated with various abnormalities, such as cardiac malformations, bronchiectasis (Kartagener syndrome), polysplenia, and genitourinary abnormalities [15]. It is therefore important to identify any such abnormalities prior to any surgical procedures to avoid vascular and organ injuries. There have been some reports describing surgical procedures in patients with malignancy associated with CSI [2–6]. Several authors have reported difficulties with surgical procedures caused by the distinctive features of CSI, and some have mentioned that preoperative 3D-CT is very useful for assessing the structures of arteries and major organs in patients with CSI [5–7]. In our case, CT showed no major vascular abnormalities.

Local resection is the standard treatment for GISTs [8–12]; original or classical LECS is considered to be a safe and useful procedure because it not only enables dissection of a tumor with minimal extra gastric wall tissue but is also suitable for all tumor locations, including proximity to the esophagogastric junction or pyloric ring [13]. Recently, many modified LECS procedures have been devised to prevent gastric contents, including tumor cells and bacteria, from being spilt into the abdominal cavity. These include inverted LECS, a combination of laparoscopic and endoscopic approaches to neoplasia with a non-exposure technique (CLEAN-NET), and non-exposed endoscopic wall-inversion surgery (NEWS) [16–18]. However, original or classical LECS is currently considered to be the standard procedure for gastric SMTs without mucosal defects because the use of modified LECS procedures is limited by several factors, including tumor size, location, and technical difficulty. To our knowledge, some cases of laparoscopic surgery for gastric cancer with associated CSI have been reported; however, we could find no reports of LECS for GIST with CSI. In published reports about CSI, some authors have described reversing the operator position during surgery to facilitate recognition of anatomical features better [2, 3]; however, do not consider this to be necessary [4–6]. We intended both to mainly dissect the structures with the operator’s left hand and to carefully approach the anatomy in terms of “medial” and “lateral” relations, rather than “left” and “right,” as Eisenberg have mentioned that the medial and lateral anatomical relations in patients with CSI are preserved [19]. Moreover, the operator could switch the adequate position to perform surgery more safely and easily because LECS with both endoscopic and laparoscopic points of view provides essential information to ensure safe and smooth procedure for surgeons. From these reasons, we had few technical difficulties with this procedure and completed local resection of our patient’s gastric SMT using the technique of LECS as usual.

In several reports about CSI, most authors emphasize not only that surgeons should pay more attention to the fundamentals of laparoscopic procedures in surgery of patients with CIS than in surgery of normal patients but also that accurate preoperative anatomic assessment and careful preoperative planning of laparoscopic procedures play a major role in surgery of patients with CSI [4–6]. We therefore believe that the most important aspect of LECS in patients with CSI is to preoperatively identify and assess any abnormalities of vascularization and anatomy by appropriate examinations.

## Conclusions

We here report uneventful resection by LECS of a GIST associated with CSI. LECS can be performed even in patients with CSI provided the anatomy and vascularization are carefully assessed preoperatively by diagnostic imaging.

## Consent

Written informed consent was obtained from the patient for publication of this case report and any accompanying images. A copy of the written consent is available for review by the Editor-in-Chief of this journal.

## Abbreviations

CSI: complete situs inversus
GIST: gastrointestinal stromal tumor
LECS: laparoscopic and endoscopic cooperative surgery
SMT: submucosal tumor
CT: computed tomography
